# Factors influencing the implementation of school interventions: a systematic review

**DOI:** 10.1093/eurpub/ckac131.119

**Published:** 2022-10-25

**Authors:** F Butscher, J Ellinger, C Mall

**Affiliations:** Department of Sport and Health Sciences, Technical University Munich, Munich, Germany

## Abstract

**Background:**

To investigate contextual factors that influence the implementation of obesity prevention interventions in school addressing children with low socioeconomic status (SES) is very important. Evidence about these factors helps to improve the implementation, which can promote the health of children with low SES and therefore reduce health inequity. We aimed to systematically identify, critically appraise and summarize the evidence on implementation of school-based interventions promoting obesity prevention for children with low SES.

**Methods:**

A systematic search in seven databases was conducted with the main inclusion criteria 1) school-based interventions and 2) age group 5-14 years. The Consolidated Framework for Implementation Research and its five domains and 39 categories was used to analyze the data deductively. If necessary, inductive sub-categories were defined within the categories. Contextual factors are assessed in the domain Outer Setting with four categories (A-D).

**Results:**

6.446 studies were screened and 16 studies fulfilled all inclusion criteria. Seven studies reported contextual factors in the four categories A. Needs & resources of parents (N = 4), B. Cosmopolitanism (N = 4), C. Peer pressure (N = 2), and D. External policy (N = 4) with seven sub-categories in total. In the following are examples for reported aspects in the sub-categories. In the sub-category D.2 Existing policy, policy in line with the intervention was a facilitator for implementation (N = 2), whereas lack of control over administrative changes and food served in cafeteria due to policy were reported as barriers (N = 2).

**Conclusions:**

Intervention research as well as applied health promotion should consider the complexity and interdependency of influencing factors for successful implementation. Albeit contextual factors are hardly changeable, they should be considered and addressed to reduce health inequity.

**Key messages:**

• More research is needed with detailed reporting of influencing factors, as detailed information is those of relevance for practice.

• Contributing to standardized analysis and reporting in implementation research by using a comprehensive framework.

